# The Vaccine That Was Never Mentioned: A Comparative Medico-Legal Analysis of the Duty to Inform and Document Preventive Immunization, with Proposed Practice Guidelines

**DOI:** 10.3390/vaccines14080664

**Published:** 2026-07-29

**Authors:** Sanit Thongsriratch, Therdpong Thongseiratch, Saratis Pairoh, Puttichart Khantee

**Affiliations:** 1 Faculty of Medicine, Prince of Songkla University, Songkhla 90110, Thailand; 2 Department of Pediatrics, Faculty of Medicine Ramathibodi Hospital, Mahidol University, Bangkok 73170, Thailand; 3 Office of the Attorney General, Bangkok 10210, Thailand

**Keywords:** vaccination, informed consent, informed refusal, medical liability, preventive medicine, standard of care, causation, clinical documentation, low- and middle-income countries, implementation, public health law

## Abstract

**Background/Objectives:** Vaccine-preventable disease may generate medico-legal disputes not only after an adverse event following immunization, but also when a clinician is alleged to have failed to mention, recommend, revisit, refer for, or document a clinically relevant vaccine. We examine this “unmentioned vaccine” problem while distinguishing existing law (lex lata) from proposed good practice (lex ferenda). **Methods:** We conducted a documented purposive narrative and comparative medico-legal review. PubMed/MEDLINE, PubMed Central, structured scholarly web searches, publicly accessible legal repositories, and official policy websites were searched from database inception through 21 July 2026 using combinations of vaccination, informed consent/refusal, failure to recommend or vaccinate, referral, documentation, negligence, causation, and LMIC terms. Authorities were selected for doctrinal relevance and jurisdictional contrast, with citation chaining. **Results:** In the selected jurisdictions, patient-centered disclosure of material risks and reasonable alternatives is recognized in differing forms. Vaccine-specific cases are sparse and fact-sensitive and do not establish a universal duty to recommend, refer, or revisit every vaccine. Provider recommendation influences uptake, but this behavioral evidence does not itself establish legal duty or causation, and direct empirical evidence linking counselling omissions to claims remains limited. We therefore present ADRR—assess, discuss and disclose, recommend or refer, and record and revisit—as proposed practice guidelines developed by the authors and presented in conceptual form, not as a validated legal or clinical standard. **Conclusions:** ADRR may support proportionate, system-level preparedness, especially in LMICs, but implementation must account for access, supply, workforce, financing, records, and legal context. Stakeholder co-design and empirical validation are required before routine adoption.

## 1. Introduction

Vaccination is one of the most effective preventive interventions in medicine, preventing millions of deaths each year and forming a core component of primary health care and the Immunization Agenda 2030 vision that everyone, everywhere, at every age should fully benefit from vaccines [1,2,3,4]. The increasing availability of vaccines against infections and cancers—including human papillomavirus (HPV), dengue, respiratory syncytial virus, meningococcal disease, herpes zoster, influenza, and COVID-19—creates new expectations for clinicians and health systems to identify eligibility, communicate recommendations, and reduce missed opportunities [5,6,7,8].

Legal and policy discussions about vaccines have traditionally focused on adverse events after immunization, product liability, mandates, exemptions, and compensation. A smaller literature addresses the inverse allegation: that a clinician failed to inform a patient that a vaccine existed, failed to recommend a clinically indicated vaccine, failed to revisit an earlier refusal, failed to refer when the vaccine was not stocked, or failed to document counselling and refusal. Direct reported litigation is uncommon but not absent. US cases involving rubella and pneumococcal vaccination show that such claims can arise, while also showing how strongly their outcome depends on the clinician–patient relationship, scope of the encounter, expert evidence, foreseeability, and causation [9,10,11].

This review uses the phrase “the vaccine that was never mentioned” to identify this preventive-liability problem. The phrase does not imply that every missed vaccination opportunity is negligence or that the duties to recommend, refer, or revisit are already settled across jurisdictions. Instead, the review separates: (i) existing disclosure and patient-information principles; (ii) context-dependent standard-of-care arguments concerning recommendation; and (iii) proposed practices concerning referral, recall, and structured documentation. Throughout the manuscript, “legal obligation” is reserved for lex lata in a specified jurisdiction, whereas “proposed practice,” “policy recommendation,” and the ADRR practice guidelines denote lex ferenda.

The issue is especially important for low- and middle-income countries (LMICs). New vaccines may enter through private markets, geographically limited programs, or risk-based initiatives before national adoption. Supply may be intermittent, public awareness uneven, financing fragmented, and documentation infrastructure variable. Missed opportunities may therefore reflect stock-outs, cost, workforce pressure, fragmented registries, inconsistent recommendations, or limited referral pathways rather than an individual clinician’s omission. Comparative legal preparedness must balance autonomy and professional responsibility with equity, feasibility, and shared health-system accountability.

The objectives of this review are to: (i) describe legal foundations relevant to vaccination counselling; (ii) compare selected common-law, European, civil-law, and hybrid approaches without treating them as internally uniform; (iii) distinguish current law from emerging interpretations and author proposals; (iv) examine vaccine-specific liability and causation scenarios; and (v) propose context-sensitive ADRR practice guidelines in explicitly conceptual form, together with a research pathway for validation.

## 2. Materials and Methods

This article is a purposive narrative and comparative medico-legal review designed to identify transferable legal principles, vaccine-specific authorities, professional guidance, empirical communication evidence, and implementation considerations. It was designed for conceptual and doctrinal synthesis rather than effect estimation, exhaustive country mapping, or jurisdiction-specific legal advice.

### 2.1. Search Strategy and Source Eligibility

The source set was re-checked and updated through 21 July 2026. Biomedical and empirical literature was identified through PubMed/MEDLINE, PubMed Central, and structured scholarly web searches. Legal authorities were identified through publicly accessible case-law databases and official court repositories, including US federal and state sources, the UK Supreme Court and BAILII, CanLII, AustLII, HUDOC, and Thai official legal sources. Policy and professional materials were retrieved from WHO, CDC/ACIP, HRSA, AAP, the General Medical Council, NICE, Immunize.org, and relevant national authorities. Reference lists and citing-authority chains were screened iteratively.

Search terms were used alone and in combination and included vaccine*, vaccination, immunization, informed consent, informed refusal, material risk, reasonable alternative, failure to recommend, failure to advise, failure to vaccinate, missed opportunity, referral, documentation, duty to warn, malpractice, negligence, standard of care, causation, no-fault compensation, LMIC, and individual jurisdiction names. Searches were supplemented with vaccine-specific terms, including HPV, pneumococcal, rubella, dengue, influenza, and COVID-19.

Sources were included when they were: (i) appellate or otherwise influential cases, statutes, or regulations directly relevant to disclosure, recommendation, refusal, third-party risk, causation, or vaccine liability; (ii) current professional guidance on vaccination counselling or documentation; (iii) systematic reviews, meta-analyses, or primary studies on provider communication and vaccine uptake; or (iv) implementation and health-system literature relevant to LMIC feasibility. Duplicate items, purely promotional materials, non-substantive commentary, and sources unrelated to clinician-level counselling or documentation were excluded. Vaccine product-injury authorities were retained only when they clarified warning, compensation, or the allocation of responsibility between manufacturers and clinicians.

### 2.2. Jurisdiction Selection and Comparative Analysis

The United States, United Kingdom, Canada, and Australia were selected because they illustrate distinct common-law approaches and contain leading authorities on disclosure, alternatives, and causation. European Court of Human Rights and selected civil-law or hybrid materials were included to contrast sources of duty, public-health proportionality, and compensation structures. Thailand was included as the authors’ home LMIC example, with statutory patient-information rights and mixed public–private vaccine access. Selection was illustrative rather than exhaustive; no claim is made that common-law or civil-law jurisdictions are internally uniform.

The synthesis was structured to combine the clinical vaccination and medico-legal expertise represented within the author team (Box 1). Each substantive proposition was categorized as: (1) lex lata—law presently in force in a specified jurisdiction; (2) a context-dependent inference from existing doctrine or professional standards; or (3) lex ferenda—an author-proposed practice or policy. Professional guidance was treated as potentially relevant to the standard of care, not as automatically creating a legal duty. No quantitative pooling was undertaken.

### 2.3. Review Limitations and Reporting Approach

Because this is a narrative and doctrinal review, it does not use a PRISMA flow diagram, duplicate independent screening, formal risk-of-bias assessment, or a comprehensive inventory of every jurisdiction. Transparency was informed by the Scale for the Assessment of Narrative Review Articles (SANRA) [12]. The synthesis is limited by English-language and public-access constraints, rapidly changing recommendations, publication and reporting bias, and the small number of reported vaccine-counselling claims.

Because law is jurisdiction-specific and rapidly evolving, the review emphasizes transferable concepts and risk-management questions rather than definitive liability rules for any one country. It is intended to support comparative scholarship, policy design, and future empirical work; it is not legal advice for an individual clinician, institution, patient, or dispute.

## 3. Legal Foundations

### 3.1. Informed Consent, Reasonable Alternatives, and Material Risk (Box 1)

The reviewed common-law jurisdictions show an autonomy-oriented trend, but they do not use identical tests. In Canterbury v. Spence, a US federal appellate court emphasized disclosure of alternatives and material risks to inform a patient’s decision [13], while US malpractice law otherwise remains largely state-specific. In Montgomery v. Lanarkshire Health Board, the UK Supreme Court required reasonable care to ensure awareness of material risks and reasonable alternatives from the patient’s perspective [14]. The UK General Medical Council similarly frames consent as dialogue about options, benefits, harms, uncertainties, and what matters to the patient [15].

Box 1 **Six legal concepts are used in this review. These short explanations are for non-legal readers and do not replace the jurisdiction-specific analysis in the text.**

  **Informed consent and informed refusal.** A patient accepts or declines after receiving sufficient, understandable information about material benefits, risks, and reasonable alternatives; the discussion and decision are documented.

  **Material risk and reasonable alternative.** Materiality is assessed under the law of the relevant jurisdiction and may depend on what a reasonable patient in that position would consider important. A reasonable alternative is an option that legally warrants discussion in the circumstances.

  **Standard of care.** The level of care expected of a reasonably competent clinician in the relevant setting; guidelines and local practice may inform, but do not automatically determine, the standard.

  **Duty and breach.** A duty identifies a legally recognized obligation to a claimant; breach asks whether the defendant fell below the applicable standard.

  **Causation.** Even if duty and breach are shown, the claimant generally must prove that the omission factually and legally caused the injury under the jurisdiction’s evidentiary test.

  **Lex lata and lex ferenda.** Lex lata describes law currently in force in a specified jurisdiction. Lex ferenda describes a proposed rule, policy, or best practice. Professional guidance may inform legal analysis without itself being law.

Australian and Canadian law developed related but distinct approaches. Rogers v. Whitaker asks whether a reasonable person in the patient’s position would attach significance to a risk, or whether the clinician knew or should have known that the particular patient would do so [16]. Reibl v. Hughes links nondisclosure to a modified objective causation inquiry concerning what a reasonable person in the plaintiff’s circumstances would have decided [17]. Historical professional-custom authorities remain relevant to breach, and the UK Supreme Court has since clarified in McCulloch that an alternative need not be discussed merely because it exists; whether it is a reasonable treatment option may involve professional judgment supported by a responsible body of medical opinion [18,19,20,21].

Applied to vaccination, these authorities support a cautious, context-specific proposition rather than a universal rule. Vaccination may be a reasonable preventive alternative to non-vaccination, delay, screening alone, or post-infection treatment when the option is clinically relevant and legally material. Materiality may depend on disease severity, patient risk, age, pregnancy, immune status, travel, occupation, local epidemiology, cost, availability, contraindications, and expressed values. Disclosure of the risk of remaining unvaccinated may follow from established informed-consent principles in some settings, but courts have not generally held that every available vaccine must be discussed at every encounter (Table 1).

### 3.2. Informed Refusal

Informed refusal is the counterpart of informed consent [22]. Patients or parents may decline vaccination, but a signed form alone does not establish a meaningful process. A contemporaneous record can show the indication, benefits, material adverse-event information, consequences of remaining unvaccinated, questions, decision, reason for deferral or refusal, access barriers, and any plan to revisit. Whether documentation is legally required, and its evidentiary weight, varies by jurisdiction and setting.

The medico-legal literature on vaccination advice is sparse but relevant. Naprawa and Reiss argue that liability analysis should distinguish defensible recommendation followed by informed refusal from an unsupported failure to recommend or active advice against vaccination [23]. Garcia and colleagues describe refusal forms and repeated counselling in US practice [24]. Reviews of vaccinating-clinician liability and COVID-19 programs show how consent, emergency measures, compensation, and professional duties interact [25,26,27]. These sources inform risk analysis, but they do not establish the incidence of claims or a uniform legal duty to re-offer vaccination (Table 1).

### 3.3. Duty to Warn and Third-Party Risk

Duties to warn third parties are exceptional and relationship-dependent. Vaccine cases involving oral polio vaccine are instructive but arise from different facts than a failure-to-recommend claim. In Tenuto v. Lederle Laboratories, a physician owed reasonable care to the parents of an infant patient regarding foreseeable effects of the infant’s treatment on close caretakers [28]. Reyes and Davis addressed manufacturer warnings and the learned intermediary doctrine in mass-immunization settings [29,30]. These cases should not be read as creating a general duty to prevent all transmission from an unvaccinated patient.

Third-party warning cases outside vaccination, including Tarasoff, Hofmann, and Reisner, demonstrate that some courts recognize limited duties to foreseeable and identifiable third parties in serious-risk settings [31,32,33]. The scope, claimant class, and triggering relationship differ markedly by jurisdiction. Their transferable lesson is therefore modest: when vulnerable household contacts or outbreak conditions are clinically relevant, communicating and recording precautionary advice may be prudent, but legal liability cannot be generalized from these authorities (Table 1).

## 4. Vaccination as Preventive Standard of Care

### 4.1. Routine Recommendation, Risk-Based Recommendation, and Shared Clinical Decision-Making

Vaccination recommendations differ in clinical strength and may have different legal significance. A routine recommendation applies to a defined group; a risk-based recommendation depends on exposures, comorbidities, occupation, travel, pregnancy, or epidemiology; and shared clinical decision-making (SCDM) calls for individualized discussion rather than a universal default [34,35]. These categories are clinical policy constructs. Their legal effect depends on jurisdiction, adoption, timing, setting, and the scope of the clinician’s role.

This distinction matters for a failure-to-recommend theory. A standard-of-care argument is generally more plausible for a clearly recommended routine vaccine than for an SCDM option, but recommendation strength alone does not establish duty or breach. For risk-based vaccines, the threshold issue is whether assessment of the relevant risk fell within the encounter. For SCDM vaccines, a reasonable process may consist of identifying eligibility, explaining individualized benefits and uncertainties, and recording the decision. A silent chart creates evidentiary ambiguity, but silence is not by itself proof of negligence.

### 4.2. Provider Recommendation as a Clinical Intervention

Provider communication is a clinically important determinant of vaccine uptake. Systematic reviews and meta-analyses of HPV vaccination associate recommendation quality with initiation, completion, and follow-through [36,37]. Presumptive or announcement-style communication, reminders, audit and feedback, and clinician training can improve uptake in some settings [38,39,40,41]. Provider hesitancy, discomfort, perceived resistance, and inadequate training can weaken recommendations [42].

These findings establish behavioral and clinical relevance, not a legal duty. Evidence that recommendation changes uptake may strengthen the plausibility of a causal narrative, but it does not prove that the individual patient would have accepted vaccination, that timely vaccination was accessible, or that the vaccine would probably have prevented the particular injury. Direct empirical evidence linking counselling omissions to complaints, claims, settlements, or judgments remains limited. The communication literature should therefore support, not be a substitute for, jurisdiction-specific legal and causal analysis.

### 4.3. Referral When the Vaccine Is Not Stocked

Health systems frequently offer some vaccines but not others, particularly in mixed public–private systems and during phased introduction. The reviewed authorities did not reveal a general cross-jurisdictional legal duty to refer whenever an indicated vaccine is unavailable on site. Referral is therefore presented here as a proportionate proposed practice: when a vaccine is clinically relevant, reliably available elsewhere, and reasonably accessible, the clinician or system may provide accurate access information and document the plan.

Any referral expectation must be proportionate to role and resources. A rural clinician cannot guarantee supply, affordability, transport, or completion. Institutions can reduce individual burden by maintaining current referral lists, displaying subsidy and stock information, assigning follow-up responsibility, and recording structural barriers. Where access is unreliable, documentation should not convert a system failure into blame directed at the clinician or family.

## 5. Comparative Legal Approaches

### 5.1. United States

US vaccination law combines public-health authority, state-specific malpractice doctrine, professional standards, federal information requirements, and compensation legislation. Jacobson, Zucht, and Prince concern state authority and child/community protection, not a general clinician duty to recommend a vaccine [43,44,45]. They therefore provide public-health context rather than direct answers to omission-based malpractice claims.

For clinicians, duties and proof vary by state, profession, and scope of the encounter. Vaccine Information Statements must be provided before administration of certain vaccines, but VIS delivery is not equivalent to a comprehensive informed-consent process and does not answer whether a vaccine had to be raised before administration was planned [46]. Guidelines may supply evidence relevant to the standard of care, while expert-qualification and clinician–patient-relationship rules can determine whether a claim reaches the merits.

US law also separates many vaccine-injury claims from omission claims. Bruesewitz concerns statutory pre-emption of certain design-defect claims for covered vaccines [47]. It does not resolve allegations that a clinician negligently failed to assess, inform, recommend, or document. No-fault compensation structures may shape trust and remedies, but they do not generally compensate for a vaccine that was never administered.

Three reported state appellate decisions directly illuminate omission-based vaccination claims. In Monusko v. Postle, a Michigan court allowed a preconception claim alleging failure to test for rubella immunity and immunize a woman who had indicated an intention to conceive; the analysis emphasized the specific preventive purpose, foreseeability, and professional standards [9]. McNulty v. McDowell declined to impose a comparable duty on materially different facts, where two contacts were directed toward avoiding rather than planning pregnancy [10]. Smith v. Pavlovich involved an allegation that PCV7 was not recommended or administered, but the claim failed because the plaintiff lacked a properly qualified expert for the advanced-practice-nursing standard and could not establish a clinician–patient relationship with the physician defendants [11]. These cases confirm that vaccine-specific litigation exists, while underscoring that they do not establish a general duty to recommend every eligible vaccine.

### 5.2. United Kingdom

In the United Kingdom, Montgomery requires reasonable care to ensure that the patient is aware of material risks and reasonable alternative or variant treatments [14]. McCulloch adds that identifying a reasonable alternative may involve professional judgment supported by a responsible body of medical opinion [21]. In vaccination, these decisions support patient-centered discussion when vaccination is a reasonable option; they do not mean that every licensed vaccine is automatically a reasonable alternative requiring disclosure.

The practical case for discussion may be stronger for a vaccine included in a national program or clearly recommended for the patient’s risk profile than for a newly licensed, privately available, or uncertain option. Materiality and reasonableness remain patient- and context-specific, including benefits, harms, uncertainty, cost, timing, contraindications, and realistic access. The General Medical Council’s dialogue-based guidance may inform professional expectations but must be applied to the clinician’s role and the encounter [15].

### 5.3. Canada

Canadian informed-consent law, especially Reibl v. Hughes, highlights causation through a modified objective test [17]. A claimant alleging failure to recommend vaccination would generally need to establish a relevant duty and breach, that a reasonable person in the plaintiff’s circumstances would have accepted the vaccine if properly informed, and that timely vaccination would probably have prevented or mitigated the injury. Canadian scholarship on dismissal of vaccine-refusing families supports meaningful information about foregoing recommended care, but does not itself establish a nationally uniform malpractice rule [48].

### 5.4. Australia

Australian law, through Rogers v. Whitaker, uses objective and patient-specific dimensions of materiality [16]. Vaccination relevance can therefore vary with residence, outbreak conditions, pregnancy, immune status, travel, occupation, and living arrangements. The doctrine supports individualized disclosure of significant options and risks; it should not be converted into a categorical obligation to list every vaccine that might be available.

### 5.5. Europe and the European Court of Human Rights

European human-rights jurisprudence frames vaccination through privacy, bodily integrity, child welfare, proportionality, and social solidarity. In Vavřička and Others v. the Czech Republic, the European Court of Human Rights upheld a statutory childhood-vaccination policy with sanctions and preschool consequences under Article 8 [49]. The case concerns state policy, not clinician malpractice, and should not be cited as direct authority for an individual duty to recommend or refer.

Civil-law systems may locate obligations in patient-rights statutes, professional codes, administrative regulation, and compensation schemes rather than case-based negligence. Practical convergence may occur around understandable information, conformity with current recommendations, and documentation, but the source, enforceability, and remedies differ. A comparable clinical workflow does not imply doctrinal equivalence.

### 5.6. Selected Civil-Law and Hybrid Approaches

International reviews of COVID-19 vaccination liability demonstrate substantial variation in liability shields, mandatory policies, professional accountability, and no-fault compensation [27]. The relevant question is therefore not whether “common-law jurisdictions” or “civil-law jurisdictions” follow one rule, but which legal source governs the particular professional, encounter, claimant, and remedy. The shared risk-management value of assessment and documentation should not be mistaken for a shared legal duty.

### 5.7. Thailand as a Lower-Middle-Income Example

Thailand illustrates how an LMIC statutory framework can contain patient-information duties without expressly creating a vaccine-specific recommendation duty. Section 8 of the National Health Act B.E. 2550 (2007) requires public-health personnel to provide health information sufficient for an appropriate service decision and recognizes refusal after information is provided [50]. The Declaration of Patients’ Rights and Preferences affirms the right to truthful, sufficient, understandable information for consent or refusal, including through representatives for minors [51]. General negligent-injury liability arises under Civil and Commercial Code Section 420 [52], and facilities are regulated under the Sanatorium Act [53].

These instruments provide a firmer lex lata basis for adequate information and informed refusal than for an affirmative duty to recommend a specific vaccine, refer elsewhere, or repeatedly revisit a decision. Those latter expectations remain context-dependent proposals unless adopted through law, professional regulation, or institutional policy. In Thailand and comparable systems, strengthening records and access pathways can build on existing information rights, but implementation should not presume that a privately available or unfunded vaccine is realistically accessible to every patient.

### 5.8. Comparative Synthesis: Convergence, Divergence, and Limits

Across the selected jurisdictions, the clearest convergence concerns patient-centered information, the importance of the clinician–patient relationship and scope of care, the possible evidentiary role of professional guidance, and the need to prove causation. Divergence remains substantial in the source of duty, materiality test, role of professional custom, treatment of third parties, expert requirements, compensation mechanisms, and available remedies.

The comparison supports a graded conclusion. Disclosure of material information and respect for informed refusal may be existing obligations in specified settings. A duty to recommend is more contingent on role, clear contemporaneous guidance, patient-specific eligibility, and local standard of care. Referral and systematic revisit are generally better characterized as proposed quality-and-safety practices. Documentation is important evidence but does not create immunity, cure inadequate counselling, or eliminate system responsibility.

## 6. Vaccine-Specific Liability Scenarios

Omission-based vaccination claims can be framed through several pathways, but reported judicial authority is limited and many claims would encounter threshold problems involving relationship, scope of care, expert evidence, and causation. The following scenarios are analytical possibilities, not predictions that liability will arise whenever a vaccine is missed (Table 2).

### 6.1. Failure to Mention Vaccine Availability

One allegation is that the patient or parent was never told that a clinically relevant vaccine existed. The legal question is not mere licensing or theoretical availability, but whether the vaccine was a reasonable option that fell within the encounter and would have been material under the applicable law. Newness, private availability, uncertain funding, contraindications, or rapidly changing policy may weaken any inference that silence constituted breach.

### 6.2. Failure to Recommend According to Guideline

A failure-to-recommend allegation is more specific: the clinician allegedly knew or should have known that the patient met a strong contemporaneous recommendation. Monusko, McNulty, and Smith show that courts may examine the preventive purpose, foreseeability, type and scope of encounter, clinician–patient relationship, professional standard, and expert proof before reaching breach [9,10,11]. A clear guideline strengthens a standard-of-care argument but does not alone establish legal duty, breach, or causation.

### 6.3. Failure to Document Refusal

Documentation is central because later accounts of counselling and refusal may conflict. ACIP guidance supports documenting discussions and informed refusal [54], and AAP and Immunize.org provide practical forms [55,56,57]. The record should reflect a real conversation rather than a signature exercise. Documentation can support evidence of the process, but it neither guarantees immunity nor shifts responsibility for cost, stock-outs, or inaccessible services to the patient.

### 6.4. Failure to Offer or Re-Offer at Subsequent Visits

Vaccine decisions are not always final. Re-offering may be clinically useful when the clinician has a continuing relationship and eligibility persists, recommendations change, access improves, or a temporary reason for deferral resolves. The reviewed authorities do not establish a universal continuing legal duty to revisit at every encounter. Recall should therefore be proportionate, assigned within the care team, and designed to avoid alert fatigue or coercion.

### 6.5. Failure to Refer When the Vaccine Is Not Stocked

A clinician may reasonably not stock every vaccine. Where an indicated vaccine is reliably obtainable elsewhere, providing accurate access information may reduce ambiguity and missed opportunities. Where availability, price, or transport is uncertain, a chart should record the structural barrier rather than implying that referral guaranteed access. This is proposed risk-management practice, not a universal duty identified across the reviewed jurisdictions.

### 6.6. Third-Party Harm from Under-Vaccination

Third-party harm raises difficult questions of duty, foreseeability, claimant identification, transmission proof, and public policy. Scholarship considers responsibility for outbreaks associated with non-vaccination [58,59], while medical-neglect and ethical literature emphasizes the limits and context-specific nature of coercive responses [60,61]. Clinicians should communicate relevant risks to vulnerable contacts without suggesting that every subsequent infection will generate third-party liability.

### 6.7. Causation and the Evidentiary Burden

Proof of omission is ordinarily insufficient. A claimant would generally need to establish a duty owed within the relevant relationship, breach of the applicable standard, factual causation, legal causation, and damage. In disclosure cases, jurisdictions differ on whether the decision counterfactual is subjective, objective, or modified objective; Reibl illustrates the importance of the reasonable person in the plaintiff’s circumstances [17]. McCulloch also demonstrates that a claim may fail on causation even where disclosure is disputed [21].

For an unmentioned vaccine, the causal chain may require proof that the patient would have accepted the vaccine; it could have been administered in time and was realistically accessible; no contraindication would have prevented administration; the vaccine covered the relevant pathogen or outcome; and vaccination would, under the jurisdiction’s standard of proof, probably have prevented or mitigated the harm. Vaccine effectiveness is probabilistic, and alternative infection sources or disease mechanisms may complicate proof. Monusko, McNulty, and Smith illustrate the fact-specific threshold and evidentiary issues [9,10,11].

## 7. Documentation and Legal Preparedness

### 7.1. EMR Prompts and Eligibility Assessment

Eligibility assessment is the first practical step, but responsibility should be distributed across the system. Immunization information systems, electronic records, and decision support can reduce missed opportunities [62,63,64,65], yet inaccurate prompts, alert fatigue, workload, and incomplete registry data can create new risk. In low-resource settings, paper checklists or structured visit stamps may serve the same evidentiary and quality-improvement function when recommendations and supply information are kept current.

### 7.2. VIS-like Information Sheets

The US VIS model illustrates how standardized information can establish a communication floor [46]. In other settings, a VIS-like sheet should supplement—not replace—individualized dialogue. It should state the disease prevented, target group, expected benefits, material adverse events, contraindications, schedule, uncertainty, cost or subsidy, realistic access points, and what to do after an adverse event. Translation, literacy, disability access, cultural adaptation, version control, and update responsibility are system requirements.

### 7.3. Refusal Forms

A refusal form is not a legal shield and may be counterproductive if used coercively. Its value depends on the underlying conversation and accurate documentation of the vaccine, disease, benefits, material risks, consequences of non-vaccination, questions, reasons, access barriers, and revisit plan. Where a patient declines to sign, the clinician may record the discussion and, where locally appropriate, a witness [55,56,57]. Forms should not be used to disguise system unavailability as patient refusal.

### 7.4. Revisit Documentation

Revisiting documentation can distinguish a considered decision from an unresolved process. Records should differentiate “not eligible,” “assessment outside encounter,” “recommended and accepted,” “recommended and declined,” “temporarily deferred,” “unavailable on site,” “referred,” “access barrier,” and “series incomplete.” Targeted recall may be useful where there is continuing care and feasible access, but blanket reminders risk alert fatigue, documentation burden, and loss of trust.

### 7.5. Proposed ADRR Practice Guidelines

**Figure 1:**
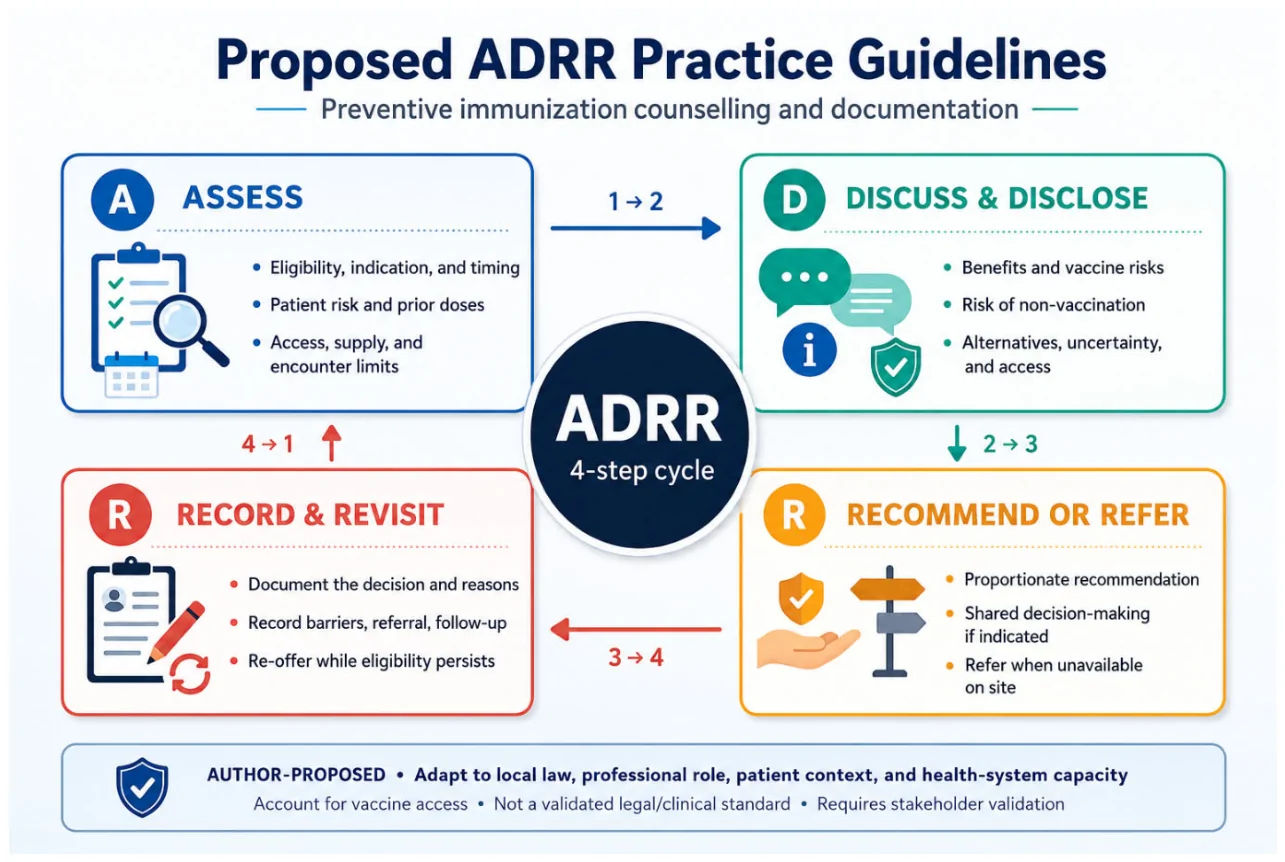
Proposed ADRR practice guidelines for preventive immunization counselling and documentation. ADRR comprises assess, discuss and disclose, recommend or refer, and record and revisit. It is an author-developed conceptual framework that should be adapted to local law, professional role, patient context, vaccine access, and health-system capacity; it is not a consensus or validated legal or clinical standard.

We propose ADRR—assess, discuss and disclose, recommend or refer, and record and revisit—as author-developed practice guidelines presented in the form of a conceptual checklist for preventive vaccine counselling and documentation. ADRR is not a consensus guideline, an evidence-validated implementation model, a legal safe harbor, or a minimum legal standard. Each element must be adapted to the clinician’s role, the strength of recommendation, local law, available resources, and realistic access. Figure 1 summarizes the four-step cycle, and Table 3 translates it into operational questions and illustrative documentation language.

### 7.6. Implementation Barriers, Safeguards, and Validation

Implementation barriers include inaccurate or absent records, intermittent vaccine supply, workforce shortages, brief encounters, limited reimbursement, fragmented responsibility, low health literacy, legal culture, privacy requirements, and the administrative cost of new documentation. Unintended consequences may include alert fatigue, defensive documentation, coercive refusal forms, inequitable referral to unaffordable services, and blame shifting from institutions to individual clinicians or families. Safeguards should include current recommendation and supply data, role clarity, minimal necessary documentation, protected time, non-punitive audit, and explicit recording of system barriers.

A validation pathway should begin with stakeholder co-design involving patients, clinicians, nurses, pharmacists, program managers, legal and ethics experts, health-information staff, and LMIC representatives. Content validity and terminology could then be tested through a transparent modified Delphi process reported against CREDES principles [66], followed by pilot studies in paper-based and digital settings. Evaluation should include acceptability, appropriateness, feasibility, fidelity, implementation cost, adoption, penetration, and sustainability [67], together with clinician workload, equity, patient trust, vaccine uptake, documentation completeness, referral completion, missed opportunities, and complaints. Until such work is completed, ADRR should be described only as a conceptual starting point.

## 8. Implications for LMICs

### 8.1. Avoiding High-Income Legal Transplants

LMIC health systems should not transplant high-income legal forms without adapting them to financing, literacy, supply, workforce, information systems, and enforcement context. A refusal form is inappropriate when the vaccine is unavailable or unaffordable, and a referral note is of limited value without a functioning destination. Legal preparedness must therefore be paired with access preparedness and with realistic allocation of responsibility across ministries, programs, facilities, and clinicians.

### 8.2. Equity and the Risk of Blame Shifting

The “unmentioned vaccine” concept can inadvertently shift responsibility from systems to clinicians or families. Under-vaccination may result from stock-outs, travel cost, fragmented registries, workforce overload, mistrust, language barriers, inconsistent national recommendations, or private-market pricing [68,69,70]. ADRR should therefore operate as a quality-improvement tool with shared accountability. Audits should distinguish failure to assess from inability to supply, refer, finance, or follow up, and should monitor whether new documentation requirements worsen disparities.

### 8.3. New Vaccines and the Private-Public Gap

New vaccines often reach LMICs unevenly: licensing, international recommendation, national scheduling, public financing, supply, and local implementation may occur at different times. HPV and dengue vaccination illustrate the need for current, product- and context-specific policy [5,6,7,8]. A clinician should not be expected to infer a legal duty from international recommendation alone when national eligibility, product indication, serostatus requirements, funding, or referral routes remain unsettled. Institutions should translate changing guidance into explicit local workflows.

### 8.4. Documentation Without Digital Infrastructure

In settings without comprehensive EMR systems, a one-line structured prompt can be more feasible than a long form. The evidentiary goal is to answer four questions without excessive burden: Was relevant eligibility assessed? Was clinically material information discussed? Was a recommendation or reliable access pathway offered when appropriate? Was the decision, barrier, and follow-up responsibility recorded? The following workflows are illustrative and unvalidated (Table 4).

Illustrative workflow A—paper-based adolescent clinic. Use a four-box stamp on the encounter sheet (assessed/discussed/accepted-declined-deferred/referred or access barrier), a centrally updated one-page referral and subsidy list, and a monthly ten-chart missed-opportunity review. Stock-outs and unaffordable cost are coded as system barriers rather than patient refusal.

Illustrative workflow B—basic digital district clinic. Use one registry-linked eligibility prompt with four outcome choices (given, declined, deferred, referred/access barrier), one optional free-text field, and a low-cost recall message only when supply and contact consent are confirmed. Prompts are suspended when recommendations or stock data are unreliable.

### 8.5. No-Fault Compensation and Trust

No-fault vaccine-injury compensation programs address eligible injuries after vaccination rather than omission-based claims, but they can support trust by acknowledging rare serious harms without requiring proof of clinician negligence [71,72,73,74,75,76,77,78]. Their design, coverage, and accessibility vary widely. Compensation should be paired with transparent counselling, adverse-event surveillance, and realistic access; it should not be presented as a substitute for informed decision-making.

Implementation can draw on adult and pediatric immunization standards, reminder evidence, digital interventions, behavioral and social-driver tools, adverse-event surveillance, and risk-communication principles [79,80,81,82,83,84,85,86,87]. Local adaptation should specify who updates recommendations and referral information, who owns follow-up, how privacy is protected, how staff time is financed, and how system barriers are audited.

## 9. Research Agenda

This review highlights four major evidence gaps. First, the incidence, disposition, and factual patterns of complaints or litigation alleging failure to recommend vaccination are largely unknown, especially outside the United States. Second, the reported cases are too sparse and context-specific to define a universal duty. Third, implementation studies seldom measure documentation completeness, referral completion, revisit rates, complaints, or legal outcomes. Fourth, LMIC research is needed on how clinicians and patients understand recommendations for licensed but unfunded or intermittently available vaccines.

Future work should proceed in stages: comparative legal mapping and claims-database studies; interviews with patients, clinicians, program managers, and risk teams; stakeholder co-design and Delphi validation of ADRR; chart audits and simulation; and pragmatic implementation trials in paper-based and digital settings. Outcomes should include acceptability, feasibility, workload, cost, equity, trust, uptake, missed opportunities, informed-refusal completeness, referral completion, and unintended consequences. The goal is safer and provides more equitable preventive care, not defensive medicine or the creation of liability by checklist.

## 10. Conclusions

The medico-legal significance of vaccination is not confined to adverse events after administration. Reported cases show that omission-based allegations can arise, but existing law remains jurisdiction-specific and fact-sensitive. The strongest cross-cutting principles concern material information, the scope of the clinician–patient relationship, the evidentiary role of professional standards, and causation. The review does not identify a universal legal duty to recommend, refer, or repeatedly revisit every vaccine.

For LMICs, a proportionate response is to strengthen counselling and records while making structural barriers visible and assigning shared system responsibility. ADRR offers author-proposed practice guidelines in conceptual form, not a validated legal or clinical standard. They should undergo multidisciplinary stakeholder consultation, content validation, feasibility testing, and outcome evaluation before routine adoption or incorporation into professional or legal standards.

## Figures and Tables

**Table 1:** Legal status and practical implications of concepts used in the “unmentioned vaccine” analysis.

Concept	Current Legal Footing in the Reviewed Authorities	Application to Vaccination
Disclosure of material risks and reasonable alternatives	Recognized in differing forms in the selected jurisdictions; tests, scope, and remedies vary.	May support discussion of disease risk, the vaccine option, and reasonable alternatives when material to this patient.
Standard of care and recommendation	Guidelines and local practice may inform breach; vaccine-specific judicial authority is sparse and fact-dependent.	A failure-to-recommend theory is most plausible where a relevant clinician–patient relationship, clear eligibility, and a strong contemporaneous recommendation are established.
Informed refusal and documentation	Professional guidance supports documentation; legal requirements and evidentiary effects vary.	Record information given, questions, decision, reason, access barriers, and any revisit plan.
Referral when a vaccine is not stocked	No general cross-jurisdictional duty was identified; predominantly a proposed proportionate practice.	Offer reliable access information when feasible and record stock, cost, or referral barriers.
Recall or revisit	Primarily quality-improvement practice or lex ferenda rather than settled law.	Use reminders where continuing care, eligibility, updated recommendations, and feasible access justify follow-up.
Third-party risk	Limited duties arise in specific relationships and jurisdictions; no general rule applies.	Address vulnerable contacts and outbreak precautions when clinically relevant.

**Table 2:** Vaccine-specific scenarios, legal uncertainties, and proportionate documentation or system responses.

Scenario	Legal or Evidentiary Issue	Proportionate Documentation or System Response
Vaccine was never mentioned	Was it a reasonable, clinically relevant option that fell within the encounter and was material under applicable law?	Record eligibility assessment, recommendation status, material access constraints, and whether the option was discussed.
Guideline-concordant vaccine was not recommended	Did the relationship, scope of care, clear eligibility, and contemporaneous standard support a recommendation?	Record the recommendation or the reason assessment/recommendation was outside the encounter; align prompts with current local guidance.
Patient or parent declined	Was the refusal informed and voluntary?	Record benefits, material risks, non-vaccination consequences, questions, decision, reason, access barriers, and any revisit plan.
Vaccine was not stocked	Was reliable referral or access information reasonably feasible?	Record on-site unavailability, known supply/cost constraints, access information offered, and who is responsible for follow-up.
Earlier refusal or deferral was not revisited	Was there continuing care, persistent eligibility, a material change, and a proportionate opportunity to revisit?	Use targeted reminders; distinguish temporary deferral, refusal, series incomplete, referral pending, and loss to follow-up.
Third-party harm was alleged	Was a duty owed to an identifiable claimant, and can transmission and preventability be proved?	Document clinically relevant advice about vulnerable contacts, outbreak precautions, and public-health referral.
Causation after vaccine-preventable disease was disputed	Would the vaccine have been accepted, administered in a timely fashion, and more likely than not have prevented or mitigated this injury?	Preserve timing, contraindication, recommendation, supply, referral, registry, and follow-up information; avoid unsupported causal conclusions.

**Table 3:** Proposed ADRR practice guidelines: operational questions and illustrative documentation language.

Step	Core Question	Illustrative Documentation Language
Assess	Does this encounter reasonably call for assessment of a routine, risk-based, catch-up, travel, occupational, pregnancy-related, or SCDM vaccine?	“HPV vaccine eligibility assessed: age 12; no prior doses; recommendation applies.”
Discuss and disclose	What benefits, material adverse-event information, non-vaccination risks, uncertainties, and alternatives matter to this patient?	“Discussed cancer prevention, schedule, common adverse events, uncertainty, and the option to vaccinate today.”
Recommend or refer	Is a strong recommendation, individualized SCDM discussion, or reliable referral appropriate and feasible?	“Recommended HPV vaccine; not stocked today; current referral and subsidy information provided.”
Record and revisit	What decision was made, what barriers exist, who owns follow-up, and when is reassessment proportionate?	“Parent deferred because of cost; access sheet provided; clinic will reassess at next scheduled visit.”

**Table 4:** Concise, context-sensitive options for staged ADRR implementation in LMIC settings.

Implementation Option	Core Workflow	Required System Safeguard
Paper minimum	Four-box assessment/counselling/outcome/access stamp; patient-held card; current referral sheet.	Updated recommendations and supply list; protected time; stock-out and cost coded as system barriers; non-punitive audit.
Basic digital	Single eligibility prompt; structured outcome; targeted recall or referral task.	Disable inaccurate alerts; confirm supply and consent before messaging; monitor alert burden, privacy, and equity.
Integrated learning system	Registry and supply links; referral completion tracking; missed-opportunity and disparity audit.	Governance, interoperability, training, reimbursement, data minimization, and iterative validation before scale-up.

## Data Availability

No new data were created or analyzed in this study. Data sharing is not applicable to this article.

## Abbreviations

ACIP: Advisory Committee on Immunization Practices; ADRR: Assess, Discuss and disclose, Recommend or Refer, Record and revisit; AAP: American Academy of Pediatrics; ECHR: European Court of Human Rights; EMR: electronic medical record; HPV: human papillomavirus; IA2030: Immunization Agenda 2030; IIS: immunization information system; LMIC: low- and middle-income country; SANRA: Scale for the Assessment of Narrative Review Articles; SCDM: shared clinical decision-making; VIS: Vaccine Information Statement; WHO: World Health Organization.
